# Implementation of the directly-georeferenced hyperspectral point cloud

**DOI:** 10.1016/j.mex.2021.101429

**Published:** 2021-06-25

**Authors:** Deep Inamdar, Margaret Kalacska, George Leblanc, J. Pablo Arroyo-Mora

**Affiliations:** a Applied Remote Sensing Laboratory, Department of Geography, McGill University, Montréal QC H3A 0B9, Canada; b Flight Research Laboratory, National Research Council of Canada, Ottawa, ON K1A 0R6, Canada

**Keywords:** Hyperspectral point cloud, Spectral data integrity, Spatial data integrity, Data fusion

## Abstract

Before pushbroom hyperspectral imaging (HSI) data can be applied in remote sensing applications, it must typically be preprocessed through radiometric correction, atmospheric compensation, geometric correction and spatial resampling procedures. After these preprocessing procedures, HSI data are conventionally given as georeferenced raster images. The raster data model compromises the spatial-spectral integrity of HSI data, leading to suboptimal results in various applications. Inamdar et al. (2021) developed a point cloud data format, the Directly-Georeferenced Hyperspectral Point Cloud (DHPC), that preserves the spatial-spectral integrity of HSI data more effectively than rasters. The DHPC is generated through a data fusion workflow that uses conventional preprocessing protocols with a modification to the digital surface model used in the geometric correction. Even with the additional elevation information, the DHPC is still stored with file sizes up to 13 times smaller than conventional rasters, making it ideal for data distribution. Our article aims to describe the DHPC data fusion workflow from Inamdar et al. (2021), providing all the required tools for its integration in pre-existing processing workflows. This includes a MATLAB script that can be readily applied to carry out the modification that must be made to the digital surface model used in the geometric correction. The MATLAB script first derives the point spread function of the HSI data and then convolves it with the digital surface model input in the geometric correction. By breaking down the MATLAB script and describing its functions, data providers can readily develop their own implementation if necessary. The derived point spread function is also useful for characterizing HSI data, quantifying the contribution of materials to the spectrum from any given pixel as a function of distance from the pixel center. Overall, our work makes the implementation of the DHPC data fusion workflow transparent and approachable for end users and data providers.

• Our article describes the Directly-Georeferenced Hyperspectral Point Cloud (DHPC) data fusion workflow, which can be readily implemented with existing processing protocols by modifying the input digital surface model used in the geometric correction.
• We provide a MATLAB function that performs the modification to the digital surface model required for the DHPC workflow. This MATLAB script derives the point spread function of the hyperspectral imager and convolves it with the digital surface model so that the elevation data are more spatially consistent with the hyperspectral imaging data as collected.
• We highlight the increased effectiveness of the DHPC over conventional raster end products in terms of spatial-spectral data integrity, data storage requirements, hyperspectral imaging application results and site exploration via virtual and augmented reality.

## Graphical Abstract

**Figure:**
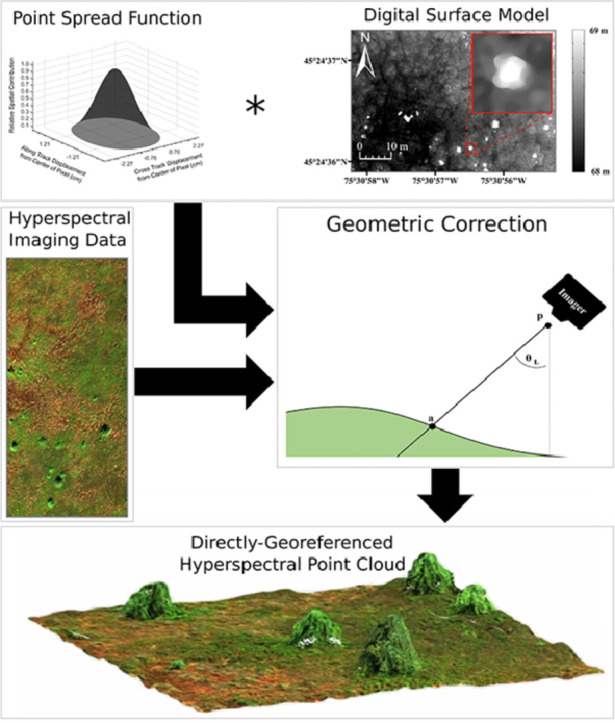

Specifications table

**Table:** 

Subject Area	Earth and Planetary Sciences
More specific subject area	Remote Sensing Data Processing
Method name	Directly-Georeferenced Hyperspectral Point Cloud
Name and reference of original method	Inamdar, D., Kalacska, M., Arroyo-Mora, J.P., & Leblanc, G. (2021). The Directly-Georeferenced Hyperspectral Point Cloud: Preserving the Integrity of Hyperspectral Imaging Data. *Frontiers in Remote Sensing, 2*, 675323
Resource availability	Required: Radiometric correction, atmospheric compensation and geometric correction software Optional: MATLAB 2020B with Hyperspectral Imaging Library from Image Processing Toolbox and CloudCompare

## Method details

### Background

Over the last four decades, the abundance of high quality spectral-spatial information captured by pushbroom hyperspectral imaging (HSI) data has been shown to be invaluable in a variety of remote sensing applications (e.g., classification, change detection, modeling, etc.) [2]. Before such HSI data products can be effectively used, they must typically undergo radiometric correction, atmospheric compensation, geometric correction and spatial resampling methodologies. The end product is a georeferenced raster image. The raster data model is the most common end product data format in HSI [3,^15^,17]. However, it is important to recognize that the raster model mispresents HSI data. For instance, hyperspectral pixels are not square, as they appear in rasters [13]. In reality, the spatial contribution to the spectrum from a single pixel is non-uniform and extends into the spatial boundaries of neighbouring pixels [5]. Furthermore, hyperspectral pixels are not uniformly distributed over the imaged scene as they appear in rasters due to various factors such as sensor design, sensor orientation and rugged terrains [16]. In raster data end products, pixels appear to be uniformly distributed due to the use of spatial resampling [12], which can compromise spatial-spectral data integrity and lead to suboptimal results in HSI applications [4].

Inamdar et al. [4] developed a point cloud HSI data representation, the Directly-Georeferenced Hyperspectral Point Cloud (DHPC), that preserves spatial-spectral data integrity more effectively than raster data end products. The DHPC is generated through a data fusion workflow that primarily uses existing processing protocols (i.e., standard radiometric correction, atmospheric compensation and geometric correction protocols). As such, it can readily be adapted and applied by data providers without large modifications to their existing processes. Our work herein first summarizes the results from Inamdar et al. [4], which substantiates the effectiveness of the DHPC over conventional square pixel rasters. Next, we describe the DHPC data fusion workflow, illustrating its similarity to conventional preprocessing workflows. We highlight a necessary modification that must be made to these conventional preprocessing workflows to generate the DHPC, providing a novel MATLAB function to carry out this step. In the following section, we break down and fully explain the MATLAB function so that data providers can readily develop their own implementation. In the final section of this work, we provide an example of the DHPC, and the intermediate data products that were used to derive it. Overall, our work aims to describe the DHPC data fusion workflow and provide all the required tools for its integration into pre-existing processing workflows.

### Method effectiveness

The effectiveness of the DHPC was substantiated over square pixel rasters by Inamdar et al. [4] based on three spatial integrity data quality metrics (i.e., pixel loss, pixel duplication and pixel shifting), data storage requirements and multiple common HSI applications (i.e., classification, spectra geo-location and target detection). The study [Inamdar et al. [4]] analyzed four different HSI datasets that were collected at three field sites with two hyperspectral sensors producing data at various spatial scales (~1.5 cm to 2.6 m). Since the spectral information is not modified in the DHPC data fusion workflow, the data product preserved spectral data integrity. Furthermore, the DHPC also preserved spatial data integrity with zero pixel loss, pixel duplication and pixel shifting. In comparison, the rasters preserved the spectral data integrity at the expense of substantial pixel loss (~50–75%) or pixel duplication (~35–75%), depending on the resampling grid resolution used in the nearest neighbour methodology. Furthermore, pixel shifting was relatively large in comparison to the DHPC, ranging from 0.33 to 1.95 pixels. In terms of data storage requirements, the DHPC had a file size that was smaller than the rasters by up to a factor of 13. In all the studied applications, the DHPC consistently outperformed the rasters. For instance, in the target detection application, false discovery and false negative rates were up to 69 % lower in the DHPC than in the studied raster datasets. Overall, the DHPC is ideal for the analysis, distribution and application of HSI data.

Although not mentioned in Inamdar et al. [4], the DHPC is also effective for site exploration via virtual (VR) and augmented (AR) reality. This is particularly useful for remote field work, which often has high logistical costs (e.g., travel, food, lodging) that limit the number of individuals that can be involved. By navigating a DHPC in VR or AR, users can study the field conditions of remote sites in a cost-effective [6] and repeatable manner [7,8]. For fragile ecosystems, VR/AR visualization of the DHPC also allows multiple users to analyze the same field site without disturbing the natural dynamics of the system. VR/AR visualization of the DHPC makes the analyzed field site more accessible for individuals that might not have the funding, time or permission to study the site firsthand. Without the structural information provided by the elevation data, the same level of immersion cannot be obtained with conventional rasters, which are viewed in two-dimensions [6,8].

### Method workflow

**Fig. 1:**
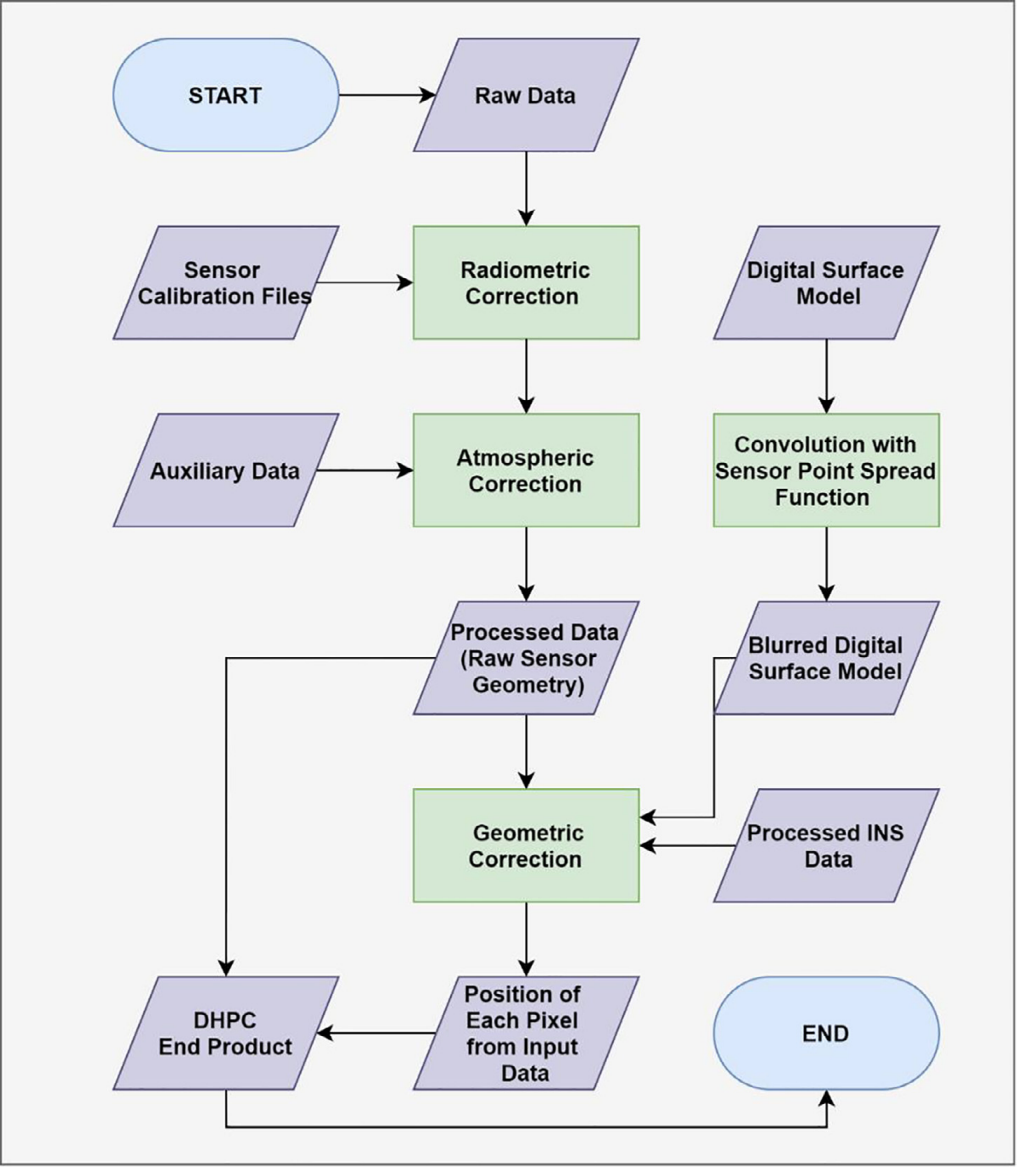
Flow chart of the hyperspectral imaging (HSI) processing workflow for the Directly-Georeferenced Hyperspectral Point Cloud (DHPC). Adapted from Inamdar et al. [4].

**Fig. 2:**
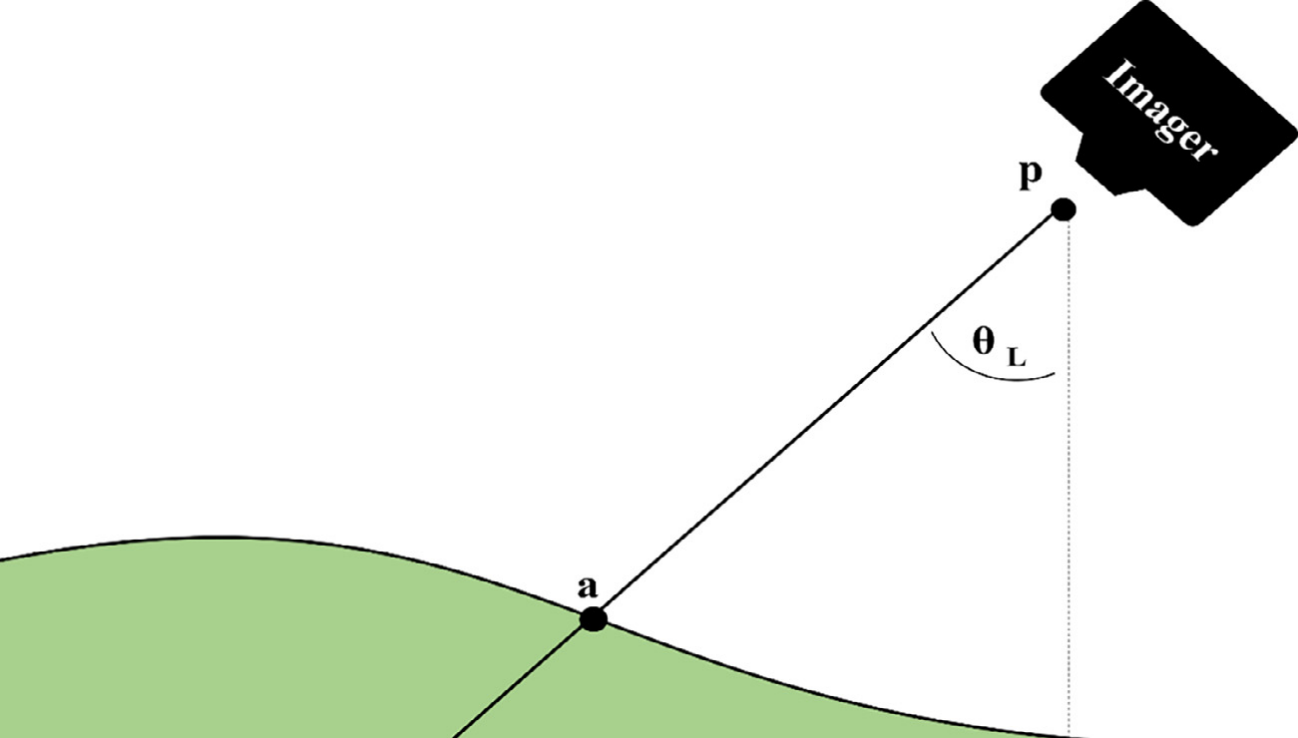
Schematic of the geometric correction. With a known sensor position (p) and look direction (θ_L_) the position of each pixel in the image space can be located in a real-world coordinate space. The pixel is located at the intersection (a) of the input digital surface model (shown in green) and a straight line that is projected at the pixel dependent look direction from the sensor position. The look direction is the angle at which incoming electromagnetic radiation is observed by any given pixel of the hyperspectral imager [10]. The look direction is calculated from the attitude, focal geometry and boresight misalignment of the sensor during data acquisition [10,18].

The data fusion workflow for the DHPC is shown in Fig. 1. In the first phase of the DHPC data fusion workflow, the input DSM is blurred through convolution with the HSI sensor point spread function (PSF). The PSF describes the spatial contribution to a single pixel of the HSI data as a function of distance from the center of the pixel [5]. As such, the convolution step makes the elevation data spatially consistent with the HSI data. After the convolution, each point in the blurred DSM corresponds to the average elevation of the objects/terrain that would contribute to a single HSI pixel. In the second phase, the radiometrically and atmospherically corrected HSI data are geometrically corrected using the blurred DSM and the inertial navigation system data of the sensor recorded during HSI data acquisition. As a result of the geometric correction, the northing, easting and elevation of each pixel of the HSI data is calculated at the intersection between the blurred DSM and a straight line that is projected from the sensor position at the pixel dependent look direction (Fig. 2). Because the blurred DSM is used in the geometric correction, each HSI pixel receives the average surface elevation of the objects/terrain contributing to it. With the projected coordinate system position (northing, easting and averaged surface elevation) the DHPC is complete. The general steps for generating the DHPC are:

1. Apply radiometric correction methodology to HSI data in raw sensor geometry (OPTIONAL).
2. Apply atmospheric compensation methodology to the HSI data from step 1 (OPTIONAL).
3. Derive PSF of the HSI data.
4. Generate blurred DSM by convolving the input DSM with the derived PSF.
5. Apply geometric correction methodology to the HSI data from step 2 using the blurred DSM.

The DHPC data fusion workflow is not limited by any particular software; data processing can be completed with the user's software of choice. Steps 1, 2 and 5 above are implemented in conventional HSI data processing workflows. As a result, data providers have pre-existing protocols to complete these steps. Steps 1 and 2 are optional because the DHPC can be used to represent the raw DN (if steps 1 and 2 are omitted) or radiance (if step 2 is omitted) data if desired. Steps 3–4 are not typically implemented in conventional data preprocessing protocols. As such, these steps represent a necessary modification that must be made to conventional preprocessing protocols to generate the DHPC. Because it can be practically difficult to derive the PSF of the input HSI data and convolve it with the input DSM, we provide a MATLAB function (see DHPC_DSM_BLUR.m in supplementary material) to carry out steps 3–4. In the following section we break down this script, describing the most important code segments and their function.

### MATLAB function

The presented MATLAB function (DHPC_DSM_BLUR.m) carries out four main tasks: 1) derive hyperspectral PSF; 2) derive convolution kernel for DSM; 3) convolve input DSM by convolution kernel; 4) output blurred DSM as ENVI standard data format. The inputs and outputs to the function are outlined in the function description:



Task 1 is completed following the derivation from Inamdar et al. [5]. In the MATLAB function, the net cross track PSF is derived by convolving the Gaussian optical PSF with the rectangular pulse detector PSF.



Next, the net along track PSF is derived by convolving the net cross track PSF by the rectangular pulse motion PSF.



With the net cross track and along track PSFs, the net PSF in 2-dimensions can be derived through vector multiplication. The two dimensions of the resultant matrix correspond with the cross track (columns) and along track (rows) displacement from the center of the pixel. To convolve the PSF with the north-oriented DSM, the 2-dimensional PSF must be rotated by the flight line heading.



To complete task 2, the input DSM must first be imported, and the pixel size must be extracted.



To derive the convolution kernel, the PSF must be spatially integrated in intervals equal to the DSM pixel size in the northing and easting direction. The kernel must also be normalized to sum to unity so that the average elevation of the convolved DSM is identical to that of the original DSM.



Afterwards, the kernel is convolved with the input DSM (task 3), blurring it based on the characteristics of the hyperspectral sensor PSF.



The final lines of the function write the blurred DSM to a new ENVI standard file (task 4). This file is saved in the same location as the original DSM. The blurred DSM is named after the original DSM, with an appended “_conv.dat”.



Below, we provide an example MATLAB code that can be used to call the MATLAB function and generate the blurred DSM.



With the blurred DSM, the DHPC data fusion workflow can be readily implemented using pre-existing processing workflows. Although there is no explicit need to output the derived PSF, it is provided by the MATLAB function to quantify the spatial contribution of the objects/terrain within any pixel. Overall, the presented MATLAB function makes the implementation of the DHPC data fusion workflow approachable for end users and data providers.

## Example dataset

Here, we provide an example of the DHPC generated in Inamdar et al. [4] from the Mer Bleue Peatland. Peatlands are important study areas due to their ability to sequester atmospheric carbon and mitigate the effects of climate change. The HSI data (see Table 1 for details) input into the DHPC data fusion workflow was collected by the µCASI-1920 hyperspectral imager (ITRES, Calagary, AB, Canada). The µCASI-1920 is a pushbroom imager that collects spectral information over 288 bands from 401 to 996 nm on a silicon-based focal plane array [1]. The DSM (0.69 cm spatial resolution) used in the data fusion workflow was generated using a Structure-from-Motion Multiview Stereo (SfM-MVS) workflow from RGB photography collected by a Canon EOS 5D Mark III equipped with a Canon EF 24–70 mm f/2.8 L II USM lens (focal length of 24 mm). In our specific implementation, the radiometric correction (step 1) was completed with proprietary software developed by the sensor manufacturer while the atmospheric correction (step 2) was carried out in ATCOR4 (as described in Soffer et al. [14]). Steps 3-4 were completed using the presented MATLAB function. The geometric correction (step 5) was completed using proprietary software developed by the sensor manufacturer. The results of the geometric correction are output to a ground coordinate look up table (GLU) that provides the easting, northing and averaged elevation of each pixel from the HSI data in its original sensor geometry. Similar outputs to the GLU are provided by other geometric correction software such as PARGE [11]. To generate the final DHPC, we compiled the GLU and HSI data (radiometrically corrected and atmospherically compensated) into a single text file. In this process, the GLU and HSI data were first imported into MATLAB as three dimensional matrices. The dimensions of the HSI matrix were 2029 by 1833 by 288 (along track pixels by cross track pixels by spectral bands) that contained the spectral information from each pixel of the HSI data. The GLU matrix was imported as a 2029 by 1833 by 3 dimensional matrix that contained the positional information (northing, easting and averaged elevation) of each pixel from the HSI data calculated during the geometric correction. The HSI and GLU matrices were then concatenated into a single 2029 by 1833 by 291 dimensional matrix. The spatial dimensions of this matrix were then flattened, creating a 2029*1833 by 291 matrix. This matrix was exported as a comma delimited text file. Due to memory limitations, this matrix was written out 1833 rows at a time, generating 2029 text files that were then merged using the Microsoft Disk Operating System (MS-DOS) copy command to generate one text file. With the position and spectral information from each pixel of the original HSI data in a single text file, the DHPC was complete.

**Table 1:** Parameters for the hyperspectral imaging data acquired over the Mer Bleue Peatland (MBP) with the µCASI-1920.

Parameter	MBP µCASI-1920 Data
Number of Cross Track Pixels	1833
Number of Along Track Pixels	2029
Sensor Field of View (°)	34.21
Nominal Flight Line Heading (° True North)	156
Nominal Altitude (m)	45
Nominal Speed (m/s)	2.7
Integration Time (ms)	9
Full width at half maximum of Optical Point Spread Function (pixels)	1.01

**Fig. 3:**
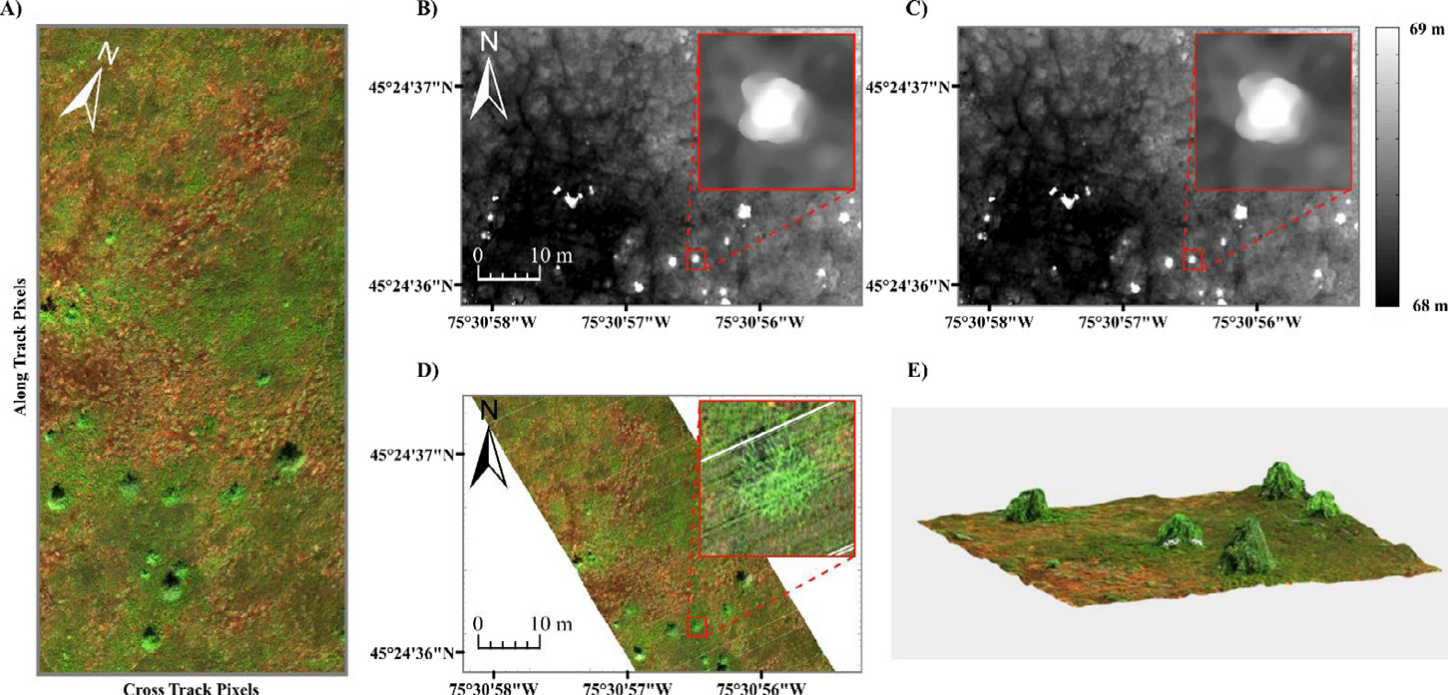
The data products used to generate the Directly-Georeferenced Hyperspectral Point Cloud (DHPC) over the Mer Bleue Peatland. A) Radiometrically and atmospherically corrected hyperspectral imaging data in raw sensor geometry from the µCASI-1920 (*R* = 639.6 nm, *G* = 550.3 nm, *B* = 459.0 nm, linearly stretched from a reflectance value of 0–12% for display purposes). B) Original digital surface elevation model (linearly stretched from an elevation value of 68–69 m). C) Blurred digital surface model (linearly stretched from an elevation value of 68–69 m). D) The DHPC viewed from above (*R* = 639.6 nm, *G* = 550.3 nm, *B* = 459.0 nm, linearly stretched from a reflectance value of 0–12% for display purposes). E) A video still of the DHPC in a 12 × 12 m region (*R* = 639.6 nm, *G* = 550.3 nm, *B* = 459.0 nm, linearly stretched from a reflectance value of 0–12% for display purposes). The full video (Supplementary_Video_1.mp4) can be found in the supplemental material. The full DHPC (HPC_288band_xyz_final.txt) can be found at https://doi.org/10.5281/zenodo.4694950.

**Fig. 4:**
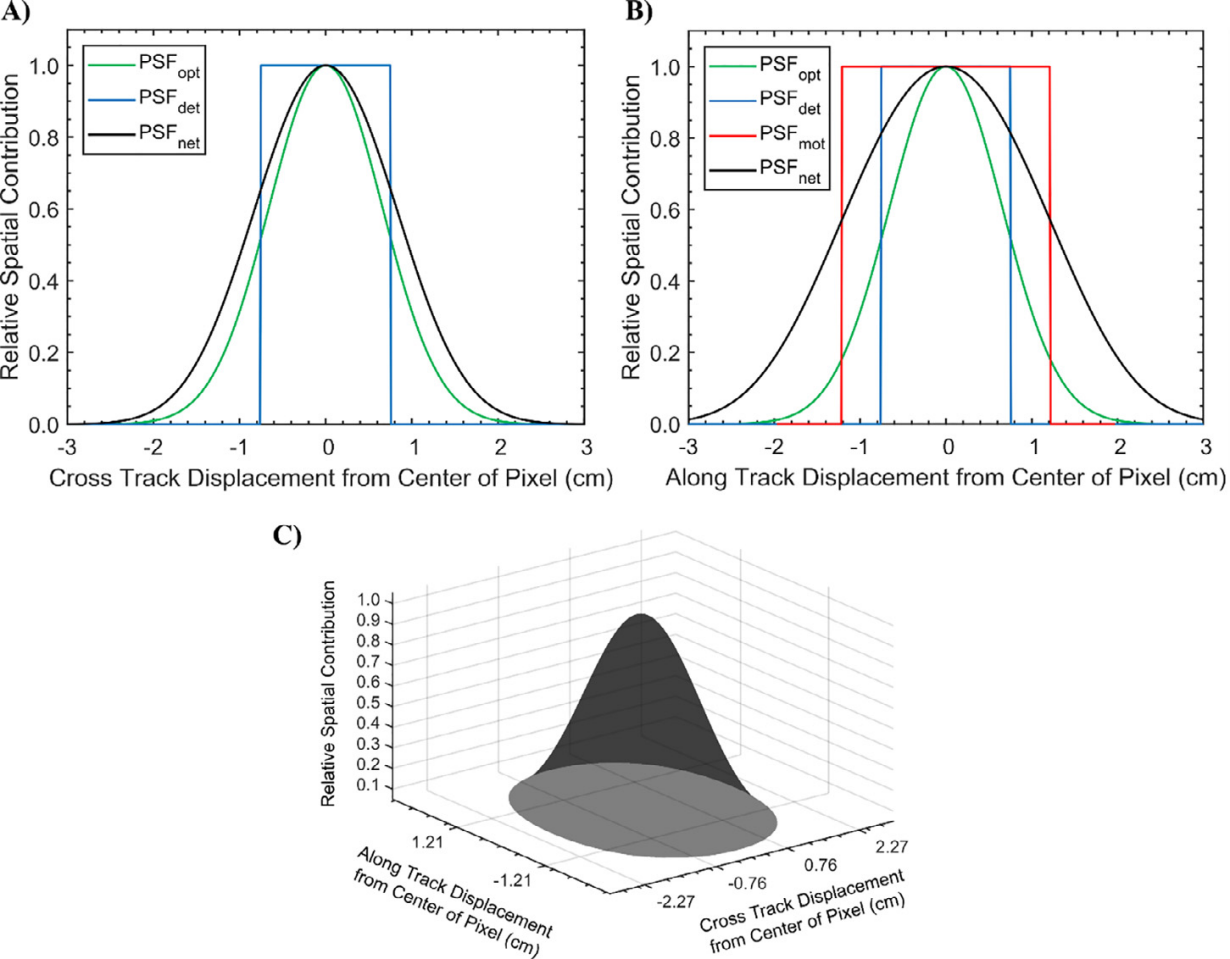
The point spread function (PSF) for the µCASI-1920 data. Panel A displays the optical PSF (PSF_opt_), detector PSF (PSF_det_) and net PSF (PSF_net_) in the cross track direction. The PSF_net_ is the convolution of the PSF_opt_ and the PSF_det_ in the cross track direction. Panel B displays the PSF_opt_, PSF_det_, motion PSF (PSF_mot_) and PSF_net_ in the along track direction. The PSF_net_ is the convolution of the PSF_mot_, PSF_opt_ and the PSF_det_ in the along track direction. Panel C displays the net PSF in both dimensions simultaneously.

The radiometrically and atmospherically corrected µCASI-1920 imagery and the input DSM can be seen in Fig. 3A and B, respectively. The PSF of the µCASI-1920 data (Fig. 4) was derived using the input parameters from Table 1 with the provided MATLAB function. The MATLAB function also convolved the derived PSF with the input DSM, generating the blurred DSM (Fig. 3C) required in the data fusion workflow. The full µCASI-1920 DHPC can be found at http://doi.org/10.5281/zenodo.4694950 (HPC_288band_xyz_final.txt). The DHPC is accompanied by a meta data file (HPC_288band_xyz_final_META.txt) that recorded important HSI data parameters such as data acquisition time, data acquisition date, sensor platform, spectral units, wavelength and full width at half maximum of each band, wavelength units, file type and map info. Fig. 3D displays the RGB bands of the DHPC, viewing the point cloud from above. A video displaying the RGB bands of the DHPC can be seen in Fig. 3E (Supplementary_Video_1.mp4).

For the purposes of site exploration, we also provide the DHPC in polygon file format (HPC_3band_xyz_shifted_final.ply in supplementary material). This file can be used to visualize the DHPC in VR and AR. It can be viewed in VR or AR at https://skfb.ly/onA6y. The polygon file format can only support three color channels. Furthermore, the variables encoded in the polygon file format must be representable as 32-bit float values. To generate the polygon file, the GLU and HSI data were first imported into MATLAB as three dimensional matrices as described above. The blue (459.0 nm), green (550.3 nm) and red (639.6 nm) bands of the HSI matrix were spectrally subset into a new 2029 by 1833 by 3 matrix. To ensure that the northing and easting values could be represented as 32-bit float values, they were centered by subtracting the minimum northing and easting value, respectively. The new HSI and GLU matrices were then concatenated, flattened and exported as a comma delimited text file (as done when generating the full DHPC as a text file). This text file was then imported into CloudCompare Stereo for conversion to polygon file format. The Mer Bleue Peatland is an ideal area for site exploration via VR and AR as the ecosystem is generally fragile and difficult to access. The DHPC allows for widespread accessibility of the site via VR and AR, allowing users to study sections of the peatland in a repeatable and cost-efficient manner [6].

In Inamdar et al. [4], the µCASI-1920 data from Mer Bleue was used to classify the hummock-hollow microtopography across the peatland. The microtopography at Mer Bleue is important to study as it covaries with surface vegetation, hydrology and carbon uptake from the atmosphere [9]. The additional elevational information provided by the DHPC led to an overall classification accuracy that was ~8% greater than the convention raster HSI datasets that contained no elevation data. This example shows the significance of the elevation information encoded in the DHPC.

## Declaration of Competing Interest

The authors declare that they have no known competing financial interests or personal relationships that could have appeared to influence the work reported in this paper.

## Appendix Supplementary materials

µCASI-1920 DHPC: HPC_288band_xyz_final.txt (http://doi.org/10.5281/zenodo.4694950) µCASI-1920 DHPC Meta Data: HPC_288band_xyz_final_META.txt (http://doi.org/10.5281/zenodo.4694950) Image, application 1

Video of DHPC: Supplementary_Video_1.mp4 HPC_3band_xyz_shifted_final.ply (also can be viewed online at https://skfb.ly/onA6y) Image, application 2

Image, video 3
